# Molecular analysis of inherited cardiomyopathy using next generation semiconductor sequencing technologies

**DOI:** 10.1186/s12967-018-1605-5

**Published:** 2018-08-30

**Authors:** Chaoxia Lu, Wei Wu, Fang Liu, Kunqi Yang, Jiacheng Li, Yaping Liu, Rongrong Wang, Nuo Si, Peng Gao, Yongtai Liu, Shuyang Zhang, Xue Zhang

**Affiliations:** 1 McKusick-Zhang Center for Genetic Medicine, State Key Laboratory of Medical Molecular Biology, Institute of Basic Medical Sciences, Chinese Academy of Medical Science & Peking Union Medical College, 5 Dong Dan San Tiao, Beijing, 100005 China; 2 Department of Cardiology, Peking Union Medical College Hospital, Chinese Academy of Medical Science & Peking Union Medical College, No. 1 Shuai Fu Yuan, Beijing, 100730 China; 3 Department of Cardiology, Fuwai Hospital, National Center for Cardiovascular Disease, Chinese Academy of Medical Sciences & Peking Union Medical College, No. 167, Beilishi Road, Beijing, 100037 China; 4 Laboratory of Clinical Genetics, Peking Union Medical College Hospital, Chinese Academy of Medical Science & Peking Union Medical College, No. 1 Shuai Fu Yuan, Beijing, 100730 China

**Keywords:** Inherited cardiomyopathy, Mutation, Next generation sequencing, *TTN*

## Abstract

**Background:**

Cardiomyopathies are the most common clinical and genetic heterogeneity cardiac diseases, and genetic contribution in particular plays a major role in patients with primary cardiomyopathies. The aim of this study is to investigate cases of inherited cardiomyopathy (IC) for potential disease-causing mutations in 64 genes reported to be associated with IC.

**Methods:**

A total of 110 independent cases or families diagnosed with various primary cardiomyopathies, including hypertrophic cardiomyopathy, dilated cardiomyopathy, restrictive cardiomyopathy, arrhythmogenic right ventricular cardiomyopathy, left ventricular non-compaction, and undefined cardiomyopathy, were collected after informed consent. A custom designed panel, including 64 genes, was screened using next generation sequencing on the Ion Torrent PGM platform. The best candidate disease-causing variants were verified by Sanger sequencing.

**Results:**

A total of 78 variants in 73 patients were identified. After excluding the variants predicted to be benign and VUS, 26 pathogenic or likely pathogenic variants were verified in 26 probands (23.6%), including a homozygous variant in the *SLC25A4* gene. Of these variants, 15 have been reported in the Human Gene Mutation Database or ClinVar database, while 11 are novel. The majority of variants were observed in the *MYH7* (8/26) and *MYBPC3* (6/26) gene. Titin (*TTN*) truncating mutations account for 13% in our dilated cardiomyopathy cases (3/23).

**Conclusions:**

This study provides an overview of the genetic aberrations in this cohort of Chinese IC patients and demonstrates the power of next generation sequencing in IC. Genetic results can provide precise clinical diagnosis and guidance regarding medical care for some individuals.

**Electronic supplementary material:**

The online version of this article (10.1186/s12967-018-1605-5) contains supplementary material, which is available to authorized users.

## Background

Cardiomyopathy is defined as the presence of a structural or functional impairment in the myocardium and is classified as either primary or secondary. Cardiomyopathy has been formally classified into five distinct forms: hypertrophic (HCM), dilated (DCM), restrictive (RCM), arrhythmogenic right ventricular cardiomyopathies (ARVC), and left ventricular non-compaction (LVNC) [1]. Genetic aberrations may contribute to a significant percentage of primary cardiomyopathy patients. Approximately 60 genes have been reported to be disease-related in inherited cardiomyopathy (IC), As such, a fast, effective genetic screening approach for IC cases would be useful. With a precise molecular diagnosis, physicians can provide accurate treatment strategies and genetic counseling for patients and their family members.

Targeted sequencing of multiple genes of interest is a rapid, cost-effective alternative to whole exome sequencing (WES) or whole genome sequencing and is becoming more commonly used in clinical laboratories. Bench-top sequencers have the advantages of low cost, flexible sequencing options, and easy-to interpret results compared to high throughput sequencers, and have significant cost and time savings over the conventional Sanger sequencing method [2].

The aim of this study was detection of pathogenic variants in 64 candidate genes associated with IC in a cohort of patients with various type of primary cardiomyopathies, and explored the potential clinical application of Ion AmpliSeq™ custom designed panel and the Ion Personal Genome Machine (PGM) system in IC.

## Methods

### Inclusion and exclusion criteria

Patients diagnosed with IC phenotypes as HCM, DCM, RCM, ARVC/D, LVNC, and overlapping or undefined phenotypes were included in this study. HCM, DCM, RCM, ARVC/D and LVNC were defined based on guidelines described by a the American Heart Association [1]. Overlapping or undefined cardiomyopathies were defined as cardiac manifestations exhibited at least two phenotypes (such as a significantly thickened interventricular septum together with left ventricular dilation) that cannot be ascribed to a single classical phenotype.

Exclusion criteria included secondary cardiomyopathies or cardiomyopathies with pathogenic mechanisms that have already been described, including inflammatory (myocarditis), stress-provoked (Tako-tsubo), peripartum, ischemic, hypertensive, valvular, hyperthyroid/hypothyroid, alcoholic, and diabetic cardiomyopathies.

### Sample collection and DNA extraction

A total of 110 unrelated patients diagnosed with IC, including HCM (n = 34), DCM (n = 22), RCM (n = 13), ARVC/D (n = 7), LVNC (n = 9), and overlapping or undefined cardiomyopathies (n = 25), were identified and enrolled at the Cardiology Clinic at the Peking Union Medical College from January 2012 to December 2015. All patients were determined to not have secondary cardiomyopathy. Of these patients, 18 had a family history of cardiomyopathy or sudden death, while 92 were sporadic cases. Clinical evaluation consisted of a medical history, family history, physical examination, 12-lead echocardiogram (ECG), transthoracic and/or transesophageal ECG, and/or cardiac magnetic resonance imaging. Peripheral blood samples were collected from patients and their family members (if available). Genomic DNA was extracted from peripheral blood leukocytes using a QIAamp DNA Blood Midi Kit (Qiagen, Hilden, Germany), according to the manufacturer’s instructions. The study was approved by the Peking Union Medical College Hospital Institutional Review Board, and all individuals signed a written informed consent.

### Panel design and library preparation

According to Online Mendelian Inheritance in Man (OMIM, http://omim.org) and PubMed literature retrieval, 64 candidate genes have been reported to be causes of inherited cardiomyopathy, and were selected for panel design (Additional file 1: Table S1). Primers of overlapping amplicons covering the coding sequence (CDS) region, untranslated regions (UTR), and flanking sequences (padding +25 base pairs) of each targeted gene were automatically generated by Ion AmpliSeq designer software. This produced 2231 amplicons, which were divided into 2 primer pools (Life Technologies; Thermo Fisher Scientific). Although Titin (*TTN*) is a major causative gene for DCM [3, 4], it was not included in the 64-gene panel. As such, we used a separate 6-gene panel (including *DMD*, *TTN*, *OBSCN*, *FBN1*, *TGFBR2* and *TGFBR1*) for 23 patients with DCM, including one patient with overlapping DCM phenotype. Amplicon libraries were prepared using the Ion AmpliSeq Library Kit v2.0 and custom designed primer pools, according to the manufacturer’s instructions. DNA fragments from different samples were ligated with barcoded sequencing adaptors using the Ion Xpress Barcode Adapter 1-16 Kit. The library was quantified with a Qubit 2.0 fluorometer (Invitrogen; Thermo Fisher Scientific).

### Next generation sequencing and data analysis

Fifteen barcoded samples were pooled in equimolar amounts. Amplified libraries were subjected to emulsion polymerase chain reaction performed on the Ion OneTouch system using the Ion PGM Hi-Q OT2 Kit. Next, ion sphere particles (ISPs) were recovered and enriched using the Ion OneTouch ES system. Enriched template-positive ISPs were sequenced on an Ion 318 V2 chip using the Ion PGM HI-Q SEQ Kit by the Ion Torrent PGM. Data from PGM runs were processed using Ion Torrent Suite 4.4 software to generate sequence reads. After sequence alignment and variant calling, synonymous variants, intronic variants far away from the exon/intron boundaries, and variants with a minor allelic frequency (MAF) ≥ 1% in the 1000 Genomes Project, the dbSNP database, and the Exome Aggregation Consortium (ExAC) database were removed from further analysis. Variants were subsequently selected according to the prevalence of each type of cardiomyopathy (for example, MAF < 0.4%, < 0.3%, < 0.2%, < 0.05% for DCM, LVNC, HCM, and ARVC, respectively) in the general population [5, 6]. NGS reads were visualized using an integrated genomic viewer (IGV).

Sequence variants were confirmed by bidirectional Sanger sequencing. Annotation of the variants was performed using ANNOVAR (http://wannovar.wglab.org/) and Seattleseq (http://snp.gs.washington.edu/SeattleSeqAnnotation137/). Polyphen2 (http://genetics.bwh.harvard.edu/pph2/), SIFT (http://sift.jcvi.org/) and MutationTaster (http://www.mutationtaster.org/) were used to predict the function of amino acid substitution. Berkeley Drosophila Genome Project (BDGP) (http://www.fruitfly.org/seq_tools/splice.html) and Human Splicing Finder (HSF) (http://www.umd.be/HSF/) were used predict the effect of intronic variants on splicing efficiency.

### Variant classification

We placed verified variants into the following categories according to guidelines from the American College of Medical Genetics and Genomics (ACMG) and Association of Molecular Pathology (AMP) [7]: pathogenic (P), likely pathogenic (LP), variant of uncertain significance (VUS), likely benign (LB) and benign (B).

## Results

### Characteristics of the Ion AmpliSeq™ custom designed panel and depth of coverage

In total, 1352 fragments with a target size of about 409.7 kb were simultaneously generate about 2231 amplicons designed based on the exons and 25 bp exon–intron boundaries of 64 cardiomyopathy-associated genes. Amplicon sizes ranged from 63 to 189 bp, with a mean amplicon length of 145 bp. The custom designed panel covered 93.73% of the bases in the target regions. For each sample, coverage of the targeted region was approximately 96.5%, with an average depth of 100. Target base coverage at 20× was above 94%, with a mean read length of 130 bp. After variant calling by Ion Torrent Suite 4.4, more than 200 variants were detected in each sample (data available upon request). After variants were filtered by allele frequency and mutation type, only zero to two variants required verification by Sanger sequencing in each sample (Additional file 2: Figure S1). All variants’ annotation information verified in this study listed in Additional file 3: Table S2.

### Mutation detection rate

We analyzed 110 patients with different primary cardiomyopathies, and detected mutations in 64 cardiomyopathy-associated genes using the NGS method. In total, 78 distinct variants from 30 genes were identified in 73 patients after filtering NGS data and verification using Sanger sequencing. Five patients had two variants in different genes (Table 2). Clinical characteristics of these patients were showed in Additional file 4: Table S3. A pathogenic or likely pathogenic variant was identified in 26 out of 110 independent cases (23.6%) (Table 1). In 45 additional patients (40.9%), VUS were found and the pathogenicity of which need further study in future (Additional file 5: Table S4). Two variants in two patients previously reported as damaging-mutation, were reclassified as B and LB. Higher detection rates of 50% (9/18) were observed in patients with a family history of cardiac disease. Among 26 pathogenic or likely pathogenic variant, 15 were recorded in HGMD or the ClinVar database and 11 were novel. These variants consisted of 15 missense, 6 nonsense, 2 frameshift, 2 splicing, and 1 stop-loss mutations.

**Table 1:** Pathogenic or likely pathogenic variants identified by cardiomyopathy NGS panels in this study

Patient No	Phenotype	Family history	Gene	Ref sequence	Mutation	Mutation Type	Genotype	HGMD /ClinVar	1000G frequency	EXAC frequency	ACMG/AMP
4	NMD +DCM	No	LMNA	NM_170707	c.810+1G>T	Splicing	HTZ	Novel/Novel	–	–	P (PVS1,PM2;PP3)
108	ARVC	No	LMNA	NM_170707	c.1400G>A, p.Trp467X	Nonsense	HTZ	Novel/Novel	–	–	P (PVS1;PM2;PP3)
8	LVNC	No	LMNA	NM_170707	c.1621C>T p.Arg541Cys	Missense	HTZ	Apical left ventricular aneurysm(DM)/Pathogenic (recurrent)	–	–	LP (PM1;PM2;PP3;PP5)
57	HCM?RCM?	No	MYBPC3	NM_000256	c.532G>A, p.Val178Met	Missense	HTZ	HCM (DM)/conflicting interpretations	–	2.05E-05	LP (PM1;PM2;PP3;PP5)
54	HCM and VT	Yes	MYBPC3	NM_000256	c.613C>T, p.Gln205Ter	Nonsense	HTZ	HCM (DM)/ Pathogenic	–	–	P (PVS1;PM2;PP3;PP5)
22	HCM	Yes	MYBPC3	NM_000256	c.772G>A, p.Glu258Lys	Missense	HTZ	HCM (DM)/Pathogenic	–	3.90E-05	P (PS3;PM1;PM2;PP3;PP5)
19	HCM	No	MYBPC3	NM_000256	c.821+1G>A	Splicing	HTZ	HCM (DM)/Pathogenic	–	4.31E-05	P (PVS1;PP5)
107	HCM	Yes	MYBPC3	NM_000256	c.2371C>T, p.Gln791Ter	Nonsense	HTZ	HCM (DM)/Likely pathogenic	–	–	P (PVS1;PM2;PP3;PP5)
45	HCM	No	MYBPC3	NM_000256	c.2827C>T, p.Arg943Ter	Nonsense	HTZ	HCM (DM)/Pathogenic	–	1.70E-05	P (PVS1;PM2;PP3;PP5)
28	HCM	No	MYH7	NM_000257	c.2146G>A, p.Gly716Arg	Missense	HTZ	HCM (DM)/P	–	–	LP (PM1;PM2;PP3;PP5)
97	HCM	No	MYH7	NM_000257	c.2332C>T, p.Asp778Asn	Missense	HTZ	Novel/Novel	–	–	LP (PM1;PM2;PP3;PP5)
10	LVNC	No	MYH7	NM_000257	c.2821C>T, p.Arg941Cys	Missense	HTZ	Novel/Novel	–	–	LP (PM1;PM2;PP2;PP3)
78	HCM	Yes	MYH7	NM_000257	c.2866G>A, p.Asp956Asn	Missense	HTZ	Novel/Novel	–	–	LP (PM1;PM2;PP2;PP3)
23	HCM	Yes	MYH7	NM_000257	c.2872G>A, p.Glu958Lys	Missense	HTZ	Novel/Novel	–	–	LP (PM1;PM2;PP2;PP3)
49	DCM	No	MYH7	NM_000257	c.3235C>T, p.Arg1079Trp	Missense	HTZ	Sudden unexpected death (DM?)/conflicting interpretations	0.0004	4.94E-05	LP (PM1;PM2;PP3;PP5;BP6)
60	RCM or HCM	Yes	MYH7	NM_000257	c.4066G>A, p.Glu1356Lys	Missense	HTZ	HCM (DM) )/LP	–	–	LP (PM1;PM2;PP2;PP3;PP5)
48	HCM	No	MYH7	NM_000257	c.4130C>T, p.Thr1377Met	Missense	HTZ	HCM (DM)/LP	–	–	LP (PM1;PM2;PP2PP3;PP5)
70	RCM HCM	No	MYL3	NM_000258	c.383G>A, p.Gly128Asp	Missense	HTZ	Novel/LP	–	–	LP (PM1;PM2;PP2;PP5)
62	HCM	Yes	PRKAG2	NM_016203.3	c.1589A>G, p.His530Arg	Missense	HTZ	HCM (DM)/P	–	–	LP (PM1;PM2;PP3;PP5)
39	Undefined cardiomyopathy	No	SLC25A4	NM_001151	c.358G>A, p.Gly120Ser	Missense	HMZ	Novel/Novel	–	–	LP (PM1;PM2;PP3;PP4)
95	HCM	Yes	TMPO	NM_003276	c.2084A>T, p.Ter695Leu	Stoploss	HTZ	Novel/Novel	–	–	LP (PM1;PM2;PM4;PP3)
65	Undefined cardiomyopathy	No	TNNC1	NM_003280	c.430A>G, p.Asn144Asp	Missense	HTZ	Novel/VUS	–	–	LP (PM1;PM2;PP2;PP5)
73	DCM	No	TNNI3	NM_000363.4	c.292C>T, p.Arg98Ter	Nonsense	HTZ	HCM(DM?)/VUS	–	9.19E-05	P (PVS1;PM2;PP3)
6	DCM	Yes	TTN	NM_133378	c.54948delC, p.Cys18316GlyfsX17	Frameshift deletion	HTZ	Novel/Novel	–	–	P (PVS1;PM2;PP1)
58	DCM	No	TTN	NM_133378	c.59644C>T,p.Gln19882Ter	Nonsense	HTZ	Novel/Novel	–	–	P (PVS1;PM2)
40	DCM?LVNC?	No	TTN	NM_133378	c.37879delG,p.Ala12627fs	Frameshift deletion	HTZ	Novel/Novel	–	–	P (PVS1;PM2)

HGMD: for human gene mutation database; DM: for damaging-mutation; VUS: uncertain significance variants; AF: atrial fibrillation; VT: ventricular tachycardia; NMD: neuromuscular disease. HMZ: homozygous; HTZ: heterozygous. B: benign; LB: likely benign; P: pathogenic; LP: likely pathogenic

**Fig. 1:**
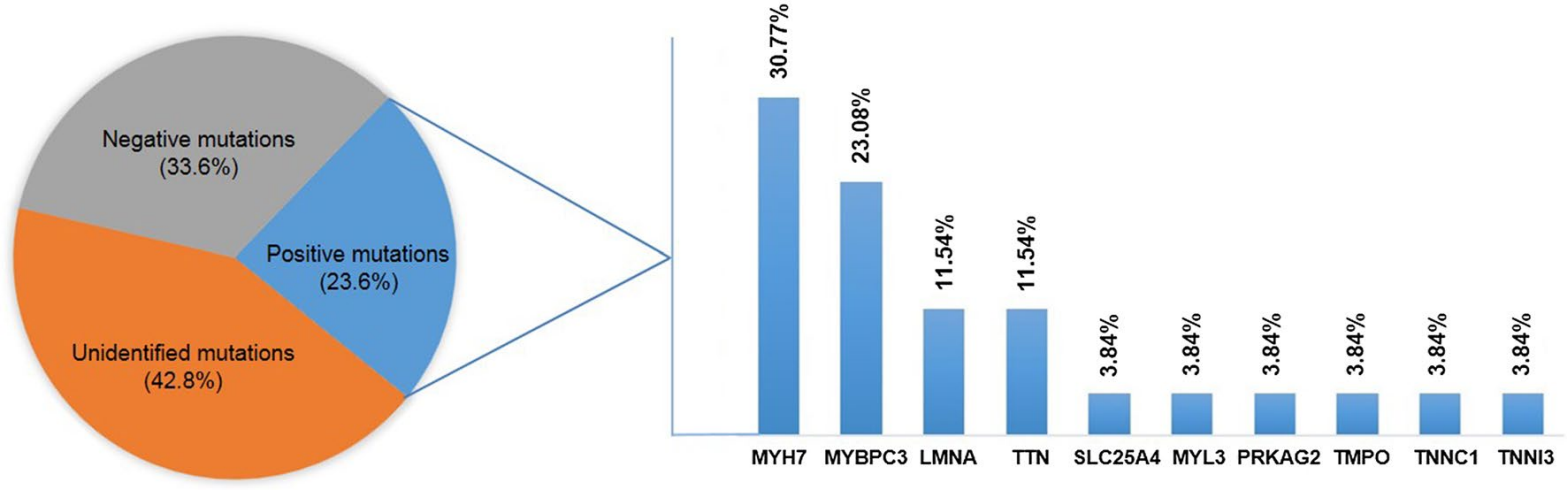
Distribution of variants across 64 genes in Chinese patients with primary cardiomyopathy. In the current cohort of Chinese patients with primary cardiomyopathy, a pathogenic or likely pathogenic mutation was confirmed in 23.6% of patients (positive mutations, blue). Mutations were found in 10 different genes, with highest mutation frequency distributed in *MYH7* and *MYBPC3*

The prevalence of disease alleles among the cardiomyopathy genes was not equally distributed. Twenty-six pathogenic or likely pathogenic mutations involved in 10 genes, and *MYH7*(8/26) and *MYBPC3* (6/26) mutations accounted for more than 50% of the variants found in cardiomyopathy cases (Fig. 1). Most of pathogenic or likely pathogenic variants were heterozygous except one homozygous pathogenic variant in the *SLC25A4* gene. All of these mutations found in our study are private, identified in a single proband. These data further confirm the genetic heterogeneity of cardiomyopathy.

### *TTN* truncating variants in DCM patients

For the consideration of sequencing cost, we put the largest causative gene (*TTN*) of DCM in a small panel and performed sequencing only in DCM patients. Of the total number of patients recruited in our study, there were 23 patients with DCM, including overlapping phenotypes. Due to the extremely large size of *TTN*, missense variants have been commonly observed in genetic screening, but no obviously frequency differences between DCM patients and healthy controls have been noted [4]. Thus, we focused on the truncating variants of *TTN* found in DCM patients. *TTN* truncations (two frameshifts and one nonsense variant, Table 1) were found in three patients, which accounted for 13% of the DCM patients in this study.

### Patients with two candidate variants

Five patients had two rare variants (Table 2). In patient 50 (*MYBPC3*: c.527C>T p.Ala176Val; *MYPN*: c.411G>C p.Arg137Ser), both variants were classified into VUS according to ACMG guidelines. In patient 56 (*BAG3*: c.772C>T p.Arg258Trp; *VCL*: c.133G>T, p.Ala45Ser), the *BAG3* variant is regarded as a damaging mutation in HGMD, but was reclassified as “likely benign” in this study based on the following criteria: BS1 (allele frequency is greater than expected for disorder) and BP6 (reputable source recently reported variant as benign, but the evidence is not available to the laboratory to perform an independent evaluation). Patient 62 (*PRKAG2*: c.1589A>G, p.His530Arg; *LAMA4*: c.241C>T p.Pro81Ser) was 26 years old and had suffered from heart failure for 2 years. Electrocardiogram showed sinus bradycardia with absolute left bundle branch block and Wolff–Parkinson–White syndrome. Echocardiography revealed hypertrophic non-obstructive cardiomyopathy, uniform thickness in the left ventricular (LV) wall, and severe LV dysfunction (EF 19%). Given that three family members, including the patient’s father, suffered sudden death, blood samples from these individuals were not available. Clinical manifestations in patient 62 were consistent with the features of *PRKAG2*-related cardiomyopathy. The missense mutation p.His530Arg in *PRKAG2* was reported as a pathogenic variant in HGMD and the ClinVar database. Although this variant likely contributed to the phenotype in patient 62, whether the *LAMA4* variant produced a “double dose” gene mutation effect will require further study. Genetic screening of first-degree relatives of the proband is important in clinical diagnosis and decision-making. In patient 87 (*RBM20*: c.3545G>A, p.Arg1182His; *SCN5A*: c.2962C>T, p.Arg988Trp), both variants were reported as damaging in HGMD. The 23-year-old patient presented with generalized cardiac enlargement with heart failure, but no signs of electrical disorders. Thus, both variants were reclassified as VUS in this patient; In patient 92 (*VCL*: c.2630C>T, p.Pro877Leu; *PSEN2*: c.998A>G, p.Glu333Gly), the *VCL* variant was likely benign because it was observed in a healthy adult individual for recessive (homozygous), dominant (heterozygous), or X-linked (hemizygous) disorders, with full penetrance expected at an early age. The pathogenicity of the *PSEN2* variant is uncertain significance.

**Table 2:** List of patients with two variants

Patient No	Phenotype	Mutation 1	Mutation 2
50	HCM	*MYBPC3*: c.527C>T,p.Ala176Val (VUS)	*MYPN*: c.411G>C,p.Arg137Ser (VUS)
56	HCM	*BAG3*: c.772C>T,p.Arg258Trp (LB)	*VCL*: c.133G>T, p.Ala45Ser (VUS)
62	HCM	*PRKAG2*: c.1589A>G,p.His530Arg (LP)	*LAMA4*: c.241C>T,p.Pro81Ser (VUS)
87	DCM	*RBM20*: c.3545G>A,p.Arg1182His (VUS)	*SCN5A*: c.2962C>T,p.Arg988Trp (VUS)
92	HCM? DCM?	*VCL*: c.2630C>T,p.Pro877Leu (LB)	*PSEN2*: c.998A>G,p.Glu333Gly (VUS)

## Discussion

More than 60 genes have been described to cause inherited cardiomyopathy. Genetic screening of these genes individually using traditional Sanger sequencing is labor-intensive and expensive. High-throughput, cost-effective genetic testing methods are urgently needed. The NGS approach provides opportunities to identify and investigate thousands of genetic aberrations simultaneously among diverse cardiomyopathy phenotypes, and is an important auxiliary tool for clinicians regarding decision-making, diagnosis, and treatment. In this study, we developed a 64-gene Ampliseq-based targeted resequencing panel, and subsequently analyzed using next generation semiconductor sequencing technology. We obtained a diagnostic yield of 23.6% (26/110). The mutation detection rate was significantly higher in familial (50%) vs. sporadic (21.7%) cardiomyopathy. Several studies applying NGS have shown different molecular diagnostic rates for different types of cardiomyopathies. For example, Cuenca et al. [8] utilized a panel of 126 genes and identified DCM-causing mutations in 73% of patients with familial dilated cardiomyopathy undergoing heart transplantation. Furthermore, Gómez et al. [9] reported a diagnostic rate of 25% when investigating 76 patients with HCM using a 9-gene panel. The divergence in diagnostic mutation rate may be ascribed to patient selection, size of panel design, and/or choice of NGS platform. In the present study, *TTN* truncating mutations were not as prevalent in patients with DCM as was previously reported, perhaps because our sample size was too small and does not represent conclusive results in the Chinese population evaluated.

High-throughput sequencing technology enable researchers to find an abundance of variants in individual cases, although determining the pathogenicity of each variant identified by NGS remains a challenge. When selecting appropriate criteria for filtering (e.g. excluding the common variants in databases, such as dbSNP and 1000 Genome), the morbidity of the disease must be considered. On the other hand, variants listed in mutation databases that may have previously been regarded as disease-causing mutations may later be proven to be benign or VUS. For example, 38 variants found in our patients were also recorded in HGMD and/or the ClinVar database. According to ACMG and AMP guidelines, only 15 variants were classified as pathogenic or likely pathogenic; 3 were reclassified as benign or likely benign, and the remaining 20 variants were reclassified as VUS (Table 1 and Additional file 5: Table S4). Thus, extreme caution needs to be used when defining a variant as disease-causing.

Forty novel single nucleotide variations (SNVs) were found in this cohort of patients that have not been reported in public mutation databases. According to ACMG guidelines, and familial co-segregation analysis, 11 novel variants were classified as pathogenic or likely pathogenic mutations (Table 1). One novel variants was classified as likely benign. The remaining 28 novel variants were classified as VUS because there was no evidence supporting classification as either pathogenic or benign.

Target sequencing of a gene panel can revise the clinical diagnosis and guidelines for management. For example, we found a homozygous *SLC25A4* (also called *ANT1*, c.358G>A, p.Gly120Ser) mutation in one patient with an HCM and DCM overlapping phenotype. The mother of the proband was a heterozygous mutation carrier and the father’s blood sample was not available. This variant was not found in the 1000 Genome, ESP6500, and ExAC databases. All three bioinformatics analyses classified this variant as damaging. A homozygous *SLC25A4* (c.368C>A, p.Ala123Asp) mutation was previously identified in a patient with mitochondrial myopathy and cardiomyopathy [10]. An in vitro study showed that the mutant produced a loss-of-function effect on SLC25A4 activity. Both of the two amino acid substitutions (p.Gly120Ser and p.Ala123Asp) occurred in conserved residues, with the position of two mutations nearby. Gly120 is located in the third transmembrane domain of ANT1 [11] within the dimerization motif “GXXXG”. This sequence is thought to be involved in high affinity association between transmembrane domains [12]. As such, we consider this to be a disease-causing mutation. A 42-year-old patient who presented with undefined cardiomyopathy had a p.Gly120Ser mutation and was born to non-consanguineous parents who were both reported to be unaffected. His cardiac manifestations included a significantly thickened interventricular septum together with left ventricular dilation and noncompaction, with subsequent development of heart failure. Histological examination of a muscle biopsy showed ragged-red fibers. Based on genetic testing results, the diagnosis was revised to mitochondrial DNA depletion syndrome 12B (cardiomyopathic type). The patient was nominated as a candidate for future heart transplantation.

There are no formal standards for classifying a variant as causative. All filtered results are based on genotype quality, frequencies, bioinformatics tools, and published data from databases. Additional criteria are needed to support or refute pathogenicity, such as in vitro functional studies or long-term follow-up during the clinical care of each patient. Familial co-segregation studies play a crucial role in determining the variants’ pathogenicity, but incomplete penetrance and variable expressivity should be considered in cardiomyopathy. The common occurrence of sudden death in cardiomyopathy made co-segregation analysis more difficult in the present study, and most variants were classified as VUS. This result still valuable, because it can provide genetic data for primary cardiomyopathies to disease-specific databases. Genetic aberrations identified in this study provided a precise clinical diagnosis, appropriate genetic counseling, and proper medical management for some IC patients with pathogenic mutation. The impacts of genetic variants on prognosis will require long-term follow-up by clinicians. Clinicians and geneticists must collaborate to determine whether a variant can be considered pathogenic following identification by NGS.

Although the rapid, economic characteristics of panel-based NGS are compelling, ampliseq-based NGS has several weaknesses. Firstly, only 94% of the target region (UTR+CDS) can be covered at the panel-design stage. Secondly, newly identified causative genes might not be included in the panel. For example, mutations in *FLNC* were reported to cause HCM and RCM after the panel was designed in this study [13, 14], so this gene was not included. Thirdly, at the PCR stage, high GC content can influence amplification efficiency. Thus, a fraction of the coding regions remains unsequenced. Moreover, false-positive rates of indel variants identified by PGM are higher than other NGS platforms, especially in homopolymer regions. In order for the application to be used in clinical testing, alternative methods must be developed to overcome these disadvantages.

Another weakness of this study is that we did not performed variants detection in healthy individuals from Chinese population in the same fashion. In public online database, such as the 1000 Genomes Project, the dbSNP database, and the Exome Aggregation Consortium (ExAC) database, the allele frequency for all candidate variants were listed, including the allele frequency in East Asian population (showed in 1000G_EAS and ExAC_EAS column in Additional file 3: Table S2). We think these data showed the allele frequency of detected variants in healthy population, to a certain extent. In order to save the cost of this study, we used the information in these databases as control.

## Conclusions

In summary, we designed an Ampliseq-based custom panel for genetic screening of IC patients and identified 26 pathogenic or likely pathogenic variants. In 45 additional IC patients, VUS were found and the pathogenicity need further confirmed. Although a relatively limited number of individuals were included in our cohort, the results provide an overview of genetic characteristics present in IC. Mutation-negative individuals from this cohort will require additional methods to discover the potential genetic defects causing their cardiomyopathy (e.g. Sanger sequencing of uncovered or low coverage region), although we cannot rule out that genetic factors are not major causes of disease in these individuals.

## Additional files

**Additional file 1: Table S1.** List of the genes selected to perform the custom panel design.

**Additional file 2: Figure S1.** Variant filtration workflow.

**Additional file 3: Table S2.** List of variant annotation information.

**Additional file 4: Table S3.** Clinical characteristics of the patients with positive variants.

**Additional file 5: Table S4.** Variants of uncertain significance or benign identified by cardiomyopathy NGS panels in this study.

## Abbreviations

ACMG: American college of medical genetics and genomics
AMP: Association of molecular pathology
ARVC: arrhythmogenic right ventricular cardiomyopathies
B: benign
BDGP: Berkeley drosophila genome project
DCM: dilated cardiomyopathies
ExAC: exome aggregation consortium database
HCM: hypertrophic cardiomyopathies
HGMD: human gene mutation database
HSF: human splicing finder
IC: inherited cardiomyopathy
IGV: integrated genomics viewer
LB: likely benign
LP: likely pathogenic
LVNC: left ventricular non-compaction
MAF: minor allele frequency
NGS: next generation sequencing
P: pathogenic
RCM: restrictive cardiomyopathies
SNVs: single nucleotide variations
TTN: titin
VUS: uncertain significance variants
WES: whole exome sequencing
