# Supplementary material for: Molecular analysis of inherited cardiomyopathy using next generation semiconductor sequencing technologies
Source: J Transl Med. 2018 Aug 30;16:241. doi: 10.1186/s12967-018-1605-5 (PMC6117967; doi:10.1186/s12967-018-1605-5)
Supplement: Supplementary file 1 — Additional file 1: Table S1. List of the genes selected to perform the custom panel design. [file 12967_2018_1605_MOESM1_ESM.doc]

SUPPLEMENTAL MATERIAL

Supplemental Table S1. List of the genes selected to perform the custom panel design

| Gene Name | Location | Transcripts ID | OMIM Gene-Phenotype Relationships | Inheritance |
| --- | --- | --- | --- | --- |
| ABCC9 | [12p12.1](http://www.omim.org/geneMap/12/207?start=-3&limit=10&highlight=207) | NM_020297 | Atrial fibrillation, familial, 12 | AD |
|  |  |  | Cardiomyopathy, dilated, 1O |  |
|  |  |  | Hypertrichotic osteochondrodysplasia | AD |
| ACTC1 | [15q14](http://www.omim.org/geneMap/15/74?start=-3&limit=10&highlight=74) | NM_005159 | Atrial septal defect 5 | AD |
|  |  |  | Cardiomyopathy, dilated, 1R | AD |
|  |  |  | Cardiomyopathy, hypertrophic, 11 | AD |
|  |  |  | Left ventricular noncompaction 4 | AD |
| ACADVL | [17p13.1](http://www.omim.org/geneMap/17/110?start=-3&limit=10&highlight=110) | NM_000018 | VLCAD deficiency | AR |
| ACTN2 | [1q43](http://www.omim.org/geneMap/1/1507?start=-3&limit=10&highlight=1507) | NM_001103 | Cardiomyopathy, dilated, 1AA, with or without LVNC | AD |
|  |  |  | Cardiomyopathy, hypertrophic, 23, with or without LVNC | AD |
| ANKRD1 | 10q23.31 | NM_014391 | a candidate for HCM | AD? |
| BAG3 | [10q26.11](http://www.omim.org/geneMap/10/516?start=-3&limit=10&highlight=516) | NM_004281 | Cardiomyopathy, dilated, 1HH | AD |
|  |  |  | Myopathy, myofibrillar, 6 | AD |
| CALR3 | [19p13.11](http://www.omim.org/geneMap/19/345?start=-3&limit=10&highlight=345) | NM_145046 | Cardiomyopathy, hypertrophic, 19 | AD |
| CAV3 | [3p25.3](http://www.omim.org/geneMap/3/24?start=-3&limit=10&highlight=24) | NM_033337 | Cardiomyopathy, familial hypertrophic | AD |
|  |  |  | Creatine phosphokinase, elevated serum | AD |
|  |  |  | Long QT syndrome 9 |  |
|  |  |  | Muscular dystrophy, limb-girdle, type IC | AR or AD |
|  |  |  | Myopathy, distal, Tateyama type | AD |
|  |  |  | Rippling muscle disease | AD |
| COX15 | [10q24.2](http://www.omim.org/geneMap/10/394?start=-3&limit=10&highlight=394) | NM_078470 | Cardioencephalomyopathy, fatal infantile, due to cytochrome coxidase deficiency 2 | AR |
|  |  |  | Leigh syndrome due to cytochrome coxidase deficiency | Mi or AR |
| CSRP3 | [11p15.1](http://www.omim.org/geneMap/11/212?start=-3&limit=10&highlight=212) | NM_003476 | Cardiomyopathy, dilated, 1M | AD |
|  |  |  | Cardiomyopathy, hypertrophic, 12 | AD |
| CTF1 | 16p11.2 | NM_001330 | a candidate for HCM | AD? |
| DES | [2q35](http://www.omim.org/geneMap/2/848?start=-3&limit=10&highlight=848) | NM_001927 | Muscular dystrophy, limb-girdle,  type 2R | AR |
|  |  |  | Cardiomyopathy, dilated, 1I  Myopathy, myofibrillar, 1 | AD |
|  |  |  |  | AR or AD |
|  |  |  | Scapuloperoneal syndrome, neurogenic, Kaeser type | AD |
| DNAJC19 | [3q26.33](http://www.omim.org/geneMap/3/724?start=-3&limit=10&highlight=724) | NM_145261 | 3-methylglutaconic aciduria, type V | AR |
| DNM1L | [12p11.21](http://www.omim.org/geneMap/12/243?start=-3&limit=10&highlight=243) | NM_012062 | Encephalopahty, lethal, due to defective mitochondrial peroxisomal fission | AD |
| DOLK | [9q34.11](http://www.omim.org/geneMap/9/468?start=-3&limit=10&highlight=468) | NM_014908 | Congenital disorder of glycosylation, type Im | AR |
| DSG2 | [18q12.1](http://www.omim.org/geneMap/18/101?start=-3&limit=10&highlight=101) | NM_001943 | Arrhythmogenic right ventricular dysplasia 10  Cardiomyopathy, dilated, 1BB | AD |
|  |  |  |  | AD |
| DSP | [6p24.3](http://www.omim.org/geneMap/6/39?start=-3&limit=10&highlight=39) | NM_004415 | Arrhythmogenic right ventricular dysplasia 8  Cardiomyopathy, dilated, with woolly hair and keratoderma  Dilated cardiomyopathy with woolly hair, keratoderma, and tooth agenesis  Epidermolysis bullosa, lethal acantholytic  Keratosis palmoplantaris striata II  Skin fragility-woolly hair syndrome | AD |
|  |  |  |  | AR |
|  |  |  |  | AD |
|  |  |  |  | AR |
|  |  |  |  |  |
|  |  |  |  | AR |
| EMD | [Xq28](http://www.omim.org/geneMap/X/790?start=-3&limit=10&highlight=790) | NM_000117 | Emery-Dreifuss muscular dystrophy 1, X-linked | XLR |
| EYA4 | [6q23.2](http://www.omim.org/geneMap/6/770?start=-3&limit=10&highlight=770) | NM_004100 | Cardiomyopathy, dilated, 1J  Deafness, autosomal dominant 10 | AD  AD |
| FHL2 | 2q12.2 | NM_201555 | a candidate for cardiomyopathy |  |
| FKTN | [9q31.2](http://www.omim.org/geneMap/9/331?start=-3&limit=10&highlight=331) | NM_001079802 | Cardiomyopathy, dilated, 1X  Muscular dystrophy-  dystroglycanopathy (congenital with brain and eye anomalies), type A, 4  Muscular dystrophy- dystroglycanopathy (congenital without mental retardation), type B, 4  Muscular dystrophy-  dystroglycanopathy (limb-girdle), type C, 4 | AR |
|  |  |  |  |  |
|  |  |  |  | AR |
|  |  |  |  | AR  AR |
| GAA | [17q25.3](http://www.omim.org/geneMap/17/845?start=-3&limit=10&highlight=845) | NM_001079804 | Glycogen storage disease II | AR |
| GATAD1 | [7q21.2](http://www.omim.org/geneMap/7/352?start=-3&limit=10&highlight=352) | NM_021167 | Cardiomyopathy, dilated, 2B | AR |
| GLA | [Xq22.1](http://www.omim.org/geneMap/X/466?start=-3&limit=10&highlight=466) | NM_000169 | Fabry disease, cardiac variant | XL |
| ILK | [11p15.4](http://www.omim.org/geneMap/11/124?start=-3&limit=10&highlight=124) | NM_004517 | dilated cardiomyopathy | AD? |
| JPH2 | [20q13.12](http://www.omim.org/geneMap/20/253?start=-3&limit=10&highlight=253) | NM_020433 | Cardiomyopathy, hypertrophic, 17 | AD |
| LAMA4 | [6q21](http://www.omim.org/geneMap/6/694?start=-3&limit=10&highlight=694) | NM_001105206 | Cardiomyopathy, dilated, 1JJ | AD |
| LAMP2 | [Xq24](http://www.omim.org/geneMap/X/571?start=-3&limit=10&highlight=571) | NM_001122606 | Danon disease | XLD |
| LDB3 | [10q23.2](http://www.omim.org/geneMap/10/305?start=-3&limit=10&highlight=305) | NM_007078 | Cardiomyopathy, dilated, 1C, with or without LVNC  Cardiomyopathy, hypertrophic, 24  Left ventricular noncompaction 3  Myopathy, myofibrillar, 4 | AD  AD  AD  AD |
| LMNA | [1q22](http://www.omim.org/geneMap/1/1021?start=-3&limit=10&highlight=1021) | NM_170707 | Cardiomyopathy, dilated, 1A  Charcot-Marie-Tooth disease, type 2B1  Emery-Dreifuss muscular dystrophy 2  Emery-Dreifuss muscular dystrophy 3  Heart-hand syndrome, Slovenian type  Hutchinson-Gilford progeria  Lipodystrophy, familial partial, 2  Malouf syndrome  Mandibuloacral dysplasia  Muscular dystrophy, congenital  Muscular dystrophy, limb-girdle, type 1B  Restrictive dermopathy, lethal | AD |
|  |  |  |  | AR |
|  |  |  |  |  |
|  |  |  |  | AD  AR |
|  |  |  |  | AD |
|  |  |  |  | AD,AR |
|  |  |  |  | AD |
|  |  |  |  | AD |
|  |  |  |  | AR |
|  |  |  |  | AD |
|  |  |  |  | AD |
|  |  |  |  | AR |
| MURC | 9q31.1 | NM_001018116 | Cardiomyopathy, dilated (HGMD) | AD |
| MYBPC3 | [11p11.2](http://www.omim.org/geneMap/11/324?start=-3&limit=10&highlight=324) | NM_000256 | Cardiomyopathy, dilated, 1MM  Cardiomyopathy, hypertrophic, 4  Left ventricular noncompaction 10 | AD |
|  |  |  |  | AD |
|  |  |  |  | AD |
| MYH6 | [14q11.2](http://www.omim.org/geneMap/14/74?start=-3&limit=10&highlight=74) | NM_002471 | Atrial septal defect 3  Cardiomyopathy, dilated, 1EE  Cardiomyopathy, hypertrophic, 14  {Sick sinus syndrome 3} | AD |
|  |  |  |  | AD |
|  |  |  |  | AD |
|  |  |  |  | ? |
| MYH7 | [14q11.2](http://www.omim.org/geneMap/14/76?start=-3&limit=10&highlight=76) | NM_000257 | Cardiomyopathy, dilated, 1S  Cardiomyopathy, hypertrophic, 1  Left ventricular noncompaction 5  Liang distal myopathy  Myopathy, myosin storage, AD Myopathy, myosin storage, AR  Scapuloperoneal syndrome, myopathic type | AD |
|  |  |  |  | AD |
|  |  |  |  | AD |
|  |  |  |  | AD |
|  |  |  |  | AD |
|  |  |  |  | AR |
|  |  |  |  | AD |
| MYL2 | [12q24.11](http://www.omim.org/geneMap/12/687?start=-3&limit=10&highlight=687) | NM_000432 | Cardiomyopathy, hypertrophic, 10 | AD |
| MYL3 | [3p21.31](http://www.omim.org/geneMap/3/213?start=-3&limit=10&highlight=213) | NM_000258 | Cardiomyopathy, hypertrophic, 8 | AD |
| MYLK2 | [20q11.21](http://www.omim.org/geneMap/20/159?start=-3&limit=10&highlight=159) | NM_033118 | Cardiomyopathy, hypertrophic, 1, digenic | AD |
| MYOZ2 | [4q26](http://www.omim.org/geneMap/4/425?start=-3&limit=10&highlight=425) | NM_016599 | Cardiomyopathy, hypertrophic, 16 | AD |
| MYPN | [10q21.3](http://www.omim.org/geneMap/10/203?start=-3&limit=10&highlight=203) | NM_032578 | Cardiomyopathy, dilated, 1KK  Cardiomyopathy, familial restrictive, 4  Cardiomyopathy, hypertrophic, 22 | AD |
|  |  |  |  | AD |
|  |  |  |  | AD |
| NDUFAF1 | [15q15.1](http://www.omim.org/geneMap/15/107?start=-3&limit=10&highlight=107) | NM_016013 | Mitochondrial complex I deficiency | Mi, XLD, AR |
| NDUFV2 | [18p11.22](http://www.omim.org/geneMap/18/42?start=-3&limit=10&highlight=42) | NM_021074 | Mitochondrial complex I deficiency | Mi, XLD, AR |
| NEBL | [10p12.31](http://www.omim.org/geneMap/10/73?start=-3&limit=10&highlight=73) | NM_006393 | a candidate for DCM | AD? |
| NEXN | [1p31.1](http://www.omim.org/geneMap/1/593?start=-3&limit=10&highlight=593) | NM_144573 | Cardiomyopathy, dilated, 1CC  Cardiomyopathy, hypertrophic, 20 | AD |
|  |  |  |  | AD |
| PDLIM3 | [4q35.1](http://www.omim.org/geneMap/4/589?start=-3&limit=10&highlight=589) | NM_014476 | a candidate for right ventricular dilated cardiomyopathy | AD? |
| PLN | [6q22.31](http://www.omim.org/geneMap/6/715?start=-3&limit=10&highlight=715) | NM_002667 | Cardiomyopathy, dilated, 1P  Cardiomyopathy, hypertrophic, 18 | AD |
|  |  |  |  | AD |
| PRKAG2 | [7q36.1](http://www.omim.org/geneMap/7/706?start=-3&limit=10&highlight=706) | NM_016203 | Cardiomyopathy, hypertrophic 6  Glycogen storage disease of heart, lethal congenital  Wolff-Parkinson-White syndrome | AD |
|  |  |  |  | AD |
|  |  |  |  | AD? |
| PSEN1 | [14q24.2](http://www.omim.org/geneMap/14/295?start=-3&limit=10&highlight=295) | NM_000021 | Acne inversa, familial, 3  Alzheimer disease, type 3  Alzheimer disease, type 3, with spastic paraparesis and apraxia  Alzheimer disease, type 3, with spastic paraparesis and unusual plaques  Cardiomyopathy, dilated, 1U  Dementia, frontotemporal  Pick disease | AD |
|  |  |  |  | AD |
|  |  |  |  | AD |
|  |  |  |  | AD |
|  |  |  |  |  |
|  |  |  |  | AD |
|  |  |  |  | AD  AD,IC |
| PSEN2 | [1q42.13](http://www.omim.org/geneMap/1/1452?start=-3&limit=10&highlight=1452) | NM_000447 | Alzheimer disease-4  Cardiomyopathy, dilated, 1V | AD |
|  |  |  |  | AD |
| RBM20 | [10q25.2](http://www.omim.org/geneMap/10/471?start=-3&limit=10&highlight=471) | NM_001134363 | Cardiomyopathy, dilated, 1DD | AD |
| SCN5A | 3p22.2 | NM_198056 | Atrial fibrillation, familial, 10  Brugada syndrome 1  Cardiomyopathy, dilated, 1E  Heart block, nonprogressive  Heart block, progressive, type IA  Long QT syndrome-3  Sick sinus syndrome 1  Ventricular fibrillation, familial, 1  {Sudden infant death syndrome, susceptibility to} | AD |
|  |  |  |  | AD |
|  |  |  |  | AD |
|  |  |  |  | AD |
|  |  |  |  | AD |
|  |  |  |  | AD |
|  |  |  |  | AR |
|  |  |  |  |  |
|  |  |  |  | AR |
| SDHA | 5p15.33 | NM_004168 | Cardiomyopathy, dilated, 1GG  Leigh syndrome  Mitochondrial respiratory chain complex II deficiency  Paragangliomas 5 | AR? |
|  |  |  |  | Mi,AR |
|  |  |  |  | AR |
|  |  |  |  |  |
|  |  |  |  | AD |
| SGCD | [5q33.2-q33.3](http://www.omim.org/geneMap/5/596?start=-3&limit=10&highlight=596) | NM_000337 | Cardiomyopathy, dilated, 1L  Muscular dystrophy, limb-girdle, type 2F | AD  AR |
| SLC25A3 | [12q23.1](http://www.omim.org/geneMap/12/610?start=-3&limit=10&highlight=610) | NM_005888 | Mitochondrial phosphate carrier deficiency | AR？ |
| SLC25A4 | [4q35.1](http://www.omim.org/geneMap/4/585?start=-3&limit=10&highlight=585) | NM_001151 | Mitochondrial DNA depletion syndrome 12 (cardiomyopathic type)  Progressive external ophthalmoplegia with mitochondrial DNA deletions 2 | AR |
|  |  |  |  | AD |
| TAZ | [Xq28](http://www.omim.org/geneMap/X/793?start=-3&limit=10&highlight=793) | NM_000116 | Barth syndrome | XLR |
| TCAP | [17q12](http://www.omim.org/geneMap/17/409?start=-3&limit=10&highlight=409) | NM_003673 | Cardiomyopathy, hypertrophic, 25  Muscular dystrophy, limb-girdle, type 2G | AD |
|  |  |  |  | AR |
| KLF10 | [8q22.3](http://www.omim.org/geneMap/8/386?start=-3&limit=10&highlight=386) | NM_005655 | hypertrophic cardiomyopathy | AD |
| TMPO | [12q23.1](http://www.omim.org/geneMap/12/609?start=-3&limit=10&highlight=609) | NM_003276 | Cardiomyopathy, dilated, 1T | AD |
| TNNC1 | [3p21.1](http://www.omim.org/geneMap/3/313?start=-3&limit=10&highlight=313) | NM_003280 | Cardiomyopathy, dilated, 1Z  Cardiomyopathy, hypertrophic, 13 | AD |
|  |  |  |  | AD |
| TNNI3 | [19q13.42](http://www.omim.org/geneMap/19/916?start=-3&limit=10&highlight=916) | NM_000363 | Cardiomyopathy, dilated, 2A  Cardiomyopathy, dilated, 1FF  Cardiomyopathy, familial restrictive, 1  Cardiomyopathy, hypertrophic, 7 | AR |
|  |  |  |  | AD |
|  |  |  |  | AD |
|  |  |  |  | AD |
| TNNT2 | [1q32.1](http://www.omim.org/geneMap/1/1285?start=-3&limit=10&highlight=1285) | NM_001001430 | Cardiomyopathy, dilated, 1D  Cardiomyopathy, familial restrictive, 3  Cardiomyopathy, hypertrophic, 2  Left ventricular noncompaction 6 | AD |
|  |  |  |  | AD |
|  |  |  |  | AD |
|  |  |  |  | AD |
| TPM1 | [15q22.2](http://www.omim.org/geneMap/15/233?start=-3&limit=10&highlight=233) | NM_000366 | Cardiomyopathy, dilated, 1Y  Cardiomyopathy, hypertrophic, 3  Left ventricular noncompaction 9 |  |
|  |  |  |  | AD |
|  |  |  |  |  |
| TTR | [18q12.1](http://www.omim.org/geneMap/18/102?start=-3&limit=10&highlight=102) | NM_000371 | Amyloidosis, hereditary, transthyretin-related  Carpal tunnel syndrome, familial  [Dystransthyretinemic hyperthyroxinemia] | AD |
|  |  |  |  | AD |
|  |  |  |  | AD |
|  |  |  | Cardiomyopathy, dilated, 1W  Cardiomyopathy, hypertrophic, 15 | AD |
| VCL | [10q22.2](http://www.omim.org/geneMap/10/269?start=-3&limit=10&highlight=269) | NM_014000 |  | AD |
|  |  |  |  |  |
