# Supplementary figures and images for: Molecular analysis of inherited cardiomyopathy using next generation semiconductor sequencing technologies
Source: J Transl Med. 2018 Aug 30;16:241. doi: 10.1186/s12967-018-1605-5 (PMC6117967; doi:10.1186/s12967-018-1605-5)

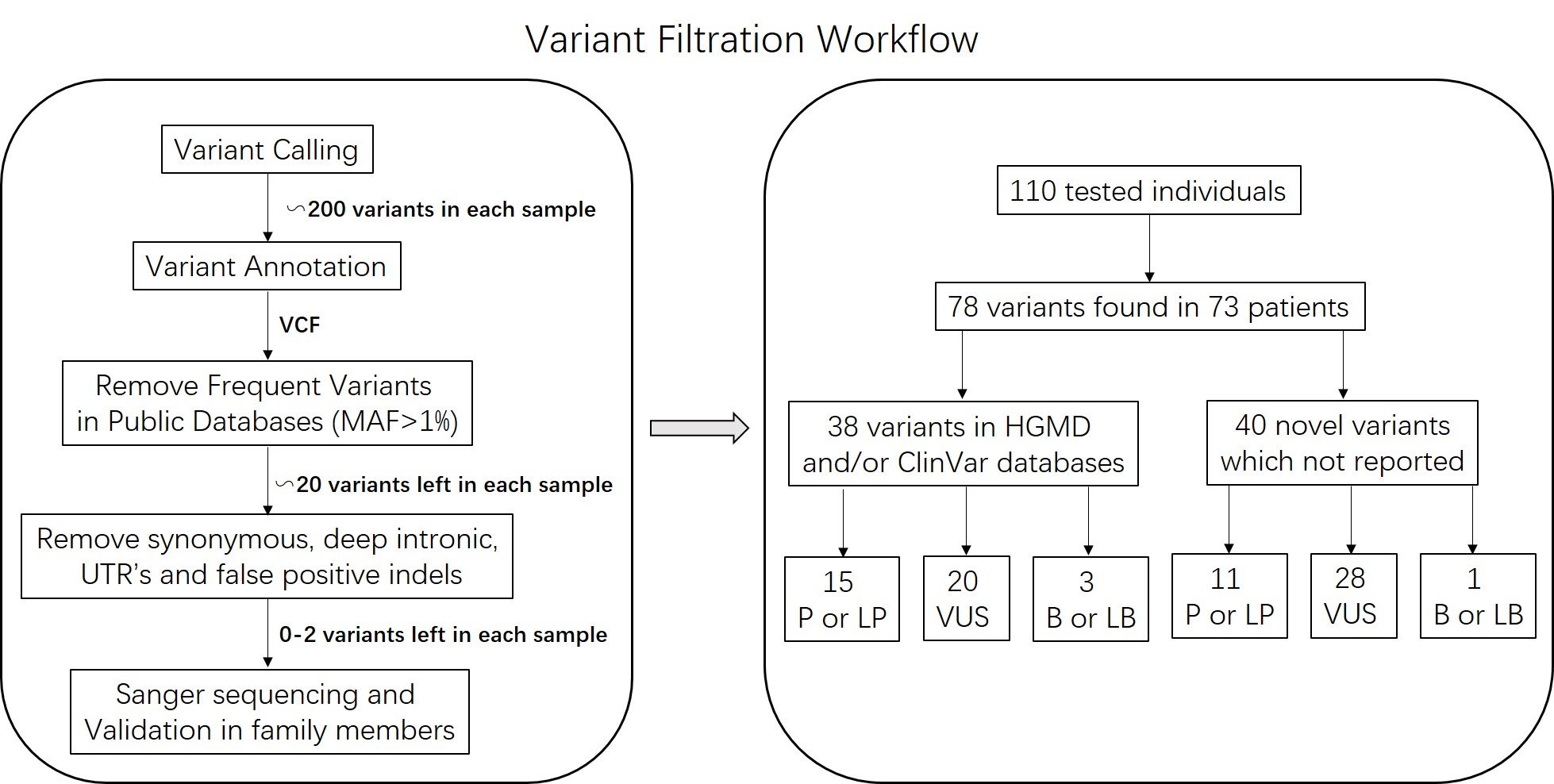

Supplement: Supplementary file 2 — Additional file 2: Figure S1. Variant filtration workflow. [file 12967_2018_1605_MOESM2_ESM.jpg]
