# Supplementary material for: Molecular analysis of inherited cardiomyopathy using next generation semiconductor sequencing technologies
Source: J Transl Med. 2018 Aug 30;16:241. doi: 10.1186/s12967-018-1605-5 (PMC6117967; doi:10.1186/s12967-018-1605-5)
Supplement: Supplementary file 4 — Additional file 4: Table S3. Clinical characteristics of the patients with positive variants. [file 12967_2018_1605_MOESM4_ESM.docx]

Table S3 Clinical characteristics of the patients with positive variants

| Pt No. | Phenotype | Echocardiogram parameters | NYHA |
| --- | --- | --- | --- |
| 100 | HCM | Asymmetric septal hypertrophy （ST=14mm）, nonobstructive | I |
| 56 | HCM | Asymmetric septal hypertrophy （ST=15mm）, nonobstructive | I |
| 25 | HCM | Asymmetric septal and apical hypertrophy (ST=17mm)，obstructive | II |
| 62 | HCM | Symmetric hypertrophy (ST=17mm), with dilated LV (LVEDd=61mm) and decreased EF (LVEF=24%) | III |
| 15 | HCM | Asymmetric septal hypertrophy （ST=28mm）, with dilated LV (LVEDd=69mm) and decreased EF (LVEF=40%) | III |
| 50 | HCM | Asymmetric septal hypertrophy （ST=18mm）, Obstructive | II |
| 22 |  | Asymmetric septal hypertrophy （ST=22mm）, Obstructive | II |
| 19 | HCM | Asymmetric septal hypertrophy （ST=19mm）, nonobstructive | III |
| 114 | HCM | Symmetric hypertrophy (ST=16mm), with dilated LV (LVEDd=59mm) , nonobstructive | III |
| 107 | HCM | Asymmetric septal hypertrophy （ST=15mm）, with dilated LV (LVEDd=58mm) and moderate decreased EF (LVEF=49%) | I |
| 83 | HCM | Asymmetric septal hypertrophy （ST=20mm）, Obstructive | II |
| 45 | HCM | Symmetric hypertrophy (ST=16mm), with moderate decreased EF （(LVEF=48%)）, nonobstructive | II |
| 28 | HCM | Asymmetric septal and apical hypertrophy (ST=22mm)，obstructive | II |
| 97 | HCM | Asymmetric septal hypertrophy （ST=19mm）, with moderate decreased EF (LVEF=50%)，obstructive | II |
| 78 | HCM | Asymmetric septal hypertrophy （ST=28mm）, obstructive | II |
| 23 | HCM | Asymmetric septal hypertrophy （ST=19mm）, nonobstructive | II |
| 48 | HCM | Asymmetric septal hypertrophy （ST=22mm）, obstructive | III |
| 90 | HCM | Asymmetric septal hypertrophy （ST=17mm）, obstructive | II |
| 38 | HCM | Asymmetric septal hypertrophy （ST=18mm）, obstructive | II |
| 95 | HCM | Asymmetric septal and apical hypertrophy (ST=20mm)，nonobstructive | I |
| 109 | HCM | Asymmetric septal and apical hypertrophy (ST=13mm)， obstructive | III |
| 79 | HCM | Asymmetric septal hypertrophy （ST=16mm）, with dilated LV (LVEDd=60mm) and decreased EF (LVEF=39%) | II |
| 61 | DCM | Four-chamber dilation (LVEDd=62mm) and decreased EF (LVEF=39%) | II |
| 68 | DCM | Dilation of LA and LV (LVEDd=66mm) and decreased EF (LVEF=24%) | III |
| 63 | DCM | LV dilation and biatrial enlargement (LVEDd=72mm) and decreased EF (LVEF=17%) | III |
| 27 | DCM | LV dilation and biatrial enlargement (LVEDd=72mm) and decreased EF (LVEF=27%) | II |
| 103 | DCM | Four-chamber dilation (LVEDd=57mm) and decreased EF (LVEF=33%) | III |
| 49 | DCM | Four-chamber dilation (LVEDd=64mm) and decreased EF (LVEF=31%) | II |
| 21 | DCM | Four-chamber dilation (LVEDd=65mm) and decreased EF (LVEF=39%) | II |
| 37 | DCM | Four-chamber dilation (LVEDd=76mm) and decreased EF (LVEF=19%) | IV |
| 67 | DCM | LV dilation and biatrial enlargement (LVEDd=59mm) and decreased EF (LVEF=31%) | III |
| 87 | DCM | Four-chamber dilation (LVEDd=65mm) and decreased EF (LVEF=25%) | III |
| 73 | DCM | Four-chamber dilation (LVEDd=72mm) and decreased EF (LVEF=11%) | IV |
| 112 | DCM | Four-chamber dilation (LVEDd=64mm) and decreased EF (LVEF=27%) | III |
| 6 | DCM | LV dilation and biatrial enlargement (LVEDd=58mm) and decreased EF (LVEF=33%) | II |
| 58 | DCM | Dilation of LA and LV (LVEDd=67mm) and decreased EF (LVEF=22%) | II |
| 93 | RCM | Biatrial enlargement, restrictive diastolic dysfunction and moderate decreased EF (LVEF=45%) | II |
| 94 | RCM | Biatrial enlargement, restrictive diastolic dysfunction and moderate decreased EF (LVEF=46%) | IV |
| 91 | RCM | Biatrial enlargement, restrictive diastolic dysfunction and normal EF (LVEF=63%) | III |
| 20 | RCM | Biatrial enlargement, restrictive diastolic dysfunction and decreased EF (LVEF=33%) | IV |
| 105 | RCM | Left atrial enlargement, diastolic dysfunction and normal EF (LVEF=74%) | III |
| 98 | RCM | Biatrial enlargement, restrictive diastolic dysfunction and decreased EF (LVEF=33%) | III |
| 80 | RCM | Biatrial enlargement, restrictive diastolic dysfunction and normal EF (LVEF=65%) | II |
| 7 | RCM | Biatrial enlargement, restrictive diastolic dysfunction and normal EF (LVEF=70%) | II |
| 11 | LVNC | Left atrial and ventricle enlargement and decreased EF (LVEF=46%). End-diastolic NC/C ratio= 2.6 | II |
| 16 | LVNC | Left atrial and ventricle enlargement and normal EF (LVEF=62%). End-diastolic NC/C ratio= 2.3 | II |
| 72 | LVNC | Four-chamber dilation and decreased EF (LVEF=33%), End-diastolic NC/C ratio= 2.7 | III |
| 88 | LVNC | Left atrial and ventricle enlargement and decreased EF (LVEF=40%). End-diastolic NC/C ratio= 2.3 | III |
| 8 | LVNC | Left ventricle enlargement and normal EF (LVEF=64%). End-diastolic NC/C ratio= 3.0 | I |
| 10 | LVNC | Biatrial and left ventricle enlargement and normal EF (LVEF=65%). End-diastolic NC/C ratio= 2.9 | II |
| 108 | ARVC | Right atrial and ventricle enlargement with decreased right ventricle EF (RVEF=35%). Normal left heart | II |
| 30 | ARVC | Right atrial and ventricle enlargement with decreased biventricle EF (RVEF=28%, LVEF=33%) | III |
| 117 | ARVC | Right ventricle enlargement with ventricular tachycardia and epsilon wave on ECG | I |
| 104 | ARVC | Right atrial and ventricle enlargement with decreased right ventricle EF (RVEF=34%). Normal left heart | II |
| 44 | undefined | Four-chamber dilation and decreased EF (LVEF=44%), Symmetric mild hypertrophy (ST=13mm) | III |
| 52 | DCM+  muscular weakness | Four-chamber dilation and decreased biventricle EF (LVEF=13%, RVEF=10%). Muscular dystrophy and weakness before heart failure | IV |
| 53 | HCM+DCM | Left atrial and ventricle enlargement with Symmetric left ventricle hypertrophy (ST=19mm) and mildly decreased EF (LVEF 50%) | II |
| 55 | myopathy+  DCM | Left atrial and ventricle enlargement with decreased EF (LVEF 27%). Muscular dystrophy and weakness before heart failure | III |
| 4 | NMD +DCM | Four-chamber dilation and decreased biventricle EF (LVEF=10%, RVEF=8%). Known neuromuscular disease before heart failure | IV |
| 54 | HCM and VT | Asymmetric septal hypertrophy （ST=18mm）, nonobstructive. Aborted sudden cardiac death caused by ventricular tachycardia | I |
| 57 | HCM？RCM？ | Biatrial enlargement and symmetric left ventricle hypertrophy (13mm)，mild decrease EF (LVEF=48%) | II |
| 60 | RCM or HCM | Biatrial enlargement and symmetric left ventricle hypertrophy (13mm)，normal EF (LVEF=58%) | I |
| 36 | DCM,  AF, VT | Biatrial and left ventricle enlargement with decreased EF (LVEF=40%), ECG recorded atrial fibrillation and ventricular tachycardia | II |
| 70 | RCM  HCM | Previously diagnosed HCM with asymmetric septal hypertrophy (18mm) and normal EF (LVEF=64%). Typical restrictive pattern was found with significant biatrial enlargement and normal left ventricle thickness 20 years later | IV |
| 47 | LVNC or DCM | Four-chamber dilation and decreased EF (LVEF=30%), End-diastolic NC/C ratio= 2.3 | III |
| 26 | DCM or LVNC | End-diastolic NC/C ratio= 2.8. Four-chamber dilation and decreased EF (LVEF=28%), | IV |
| 39 | HCM or DCM or LVNC | Left atrial and ventricle enlargement with decreased EF (LVEF 21%). symmetric left ventricle hypertrophy (13mm) and End-diastolic NC/C ratio= 2.4 | III |
| 65 | undefined | Normal left ventricle size with decreased EF (LVEF=31%), normal ventricular thickness | III |
| 116 | undefined | Mild left ventricle enlargement (LVEDd=56mm) and decreased EF (LVEF 40%), with significant late gadolinium enhancement in magnetic resonance | II |
| 40 | DCM？LVNC？ | Left atrial and ventricle enlargement with decreased EF (LVEF 24%), End-diastolic NC/C ratio= 2.5 | III |
| 66 | DCM，muscular myopathy | Left ventricle enlargement (LVEDd=60mm) and decreased EF (LVEF 46%), Muscular dystrophy and weakness before heart failure | I |
| 92 | HCM?DCM? | Left ventricle enlargement (LVEDd=58mm) and mildly decreased EF (LVEF=49%), asymmetric septal hypertrophy (15mm) | II |
| 64 | undefined | Left arial and ventricle enlargement (LVEDd=71mm) and decreased EF (LVEF= 35%), and symmetric left ventricle hypertrophy (13mm) | I |

Note: NYHA, New York Heart Association classification; LV, left ventricular; EF, Ejection fraction; LVEF, left ventricular ejection fraction; RVEF, right ventricle EF; LVEDd, left ventricular end diastolic dimension; ST, septal thickness.
