# Supplementary material for: Molecular analysis of inherited cardiomyopathy using next generation semiconductor sequencing technologies
Source: J Transl Med. 2018 Aug 30;16:241. doi: 10.1186/s12967-018-1605-5 (PMC6117967; doi:10.1186/s12967-018-1605-5)
Supplement: Supplementary file 5 — Additional file 5: Table S4. Variants of uncertain significance or benign identified by cardiomyopathy NGS panels in this study. [file 12967_2018_1605_MOESM5_ESM.doc]

**Table S4 Variants of uncertain significance identified by cardiomyopathy NGS panels in this study**

| Patient No | Phenotype | Family history | Gene | Ref sequence | Mutation | Mutation Type | Genotype | HGMD  ／ClinVar | 1000G frequency | EXAC  frequency | ACMG/AMP |
| --- | --- | --- | --- | --- | --- | --- | --- | --- | --- | --- | --- |
| 100 | HCM | No | ACTN2 | NM_001103.3 | c.1342G>A p.Glu448Lys | Missense | HTZ | Novel/VUS | . | . | VUS  (PP3) |
| 25 | HCM | No | JPH2 | NM_020433 | c.989G>A p.Thr330Met | Missense | HTZ | Novel/Novel | . | . | VUS  (PM2) |
| 62 | HCM | Yes | LAMA4 | NM_002290 | c.241C>T p.Pro81Ser | Missense | HTZ | Novel/Novel | . | . | VUS  (PM1;PM2;PP3) |
| 15 | HCM | No | LAMA4 | NM_002290 | c.4279A>G p.Lys1427Glu | Missense | HTZ | Novel/Novel | . | . | VUS  (PM2) |
| 50 | HCM | No | MYBPC3 | NM_000256 | c.527C>T p.Ala176Val | Missense | HTZ | Novel/Novel | . | 1.04E-05 | VUS  (PM1;PM2) |
|  |  |  | MYPN | NM_032578.3 | c.411G>C p.Arg137Ser | Missense | HTZ | Novel/VUS | . | 3.30E-05 | VUS |
| 83 | HCM | Yes | MYBPC3 | NM_000256 | c.2459G>A, p.Arg820Gln | Missense | HTZ | HCM (DM)/ conflicting interpretations | . | 1.66E-05 | VUS  (PM1;PM2;PP5) |
| 90 | HCM | No | MYPN | NM_032578.3 | c.832A>G, p.Arg278Gly | Missense | HTZ | Novel/Novel | . | 8.33E-06 | VUS  (PM1) |
| 38 | HCM | No | NEBL | NM_006393 | c.389A>C, p.His130Pro | Missense | HTZ | Novel/Novel | . | . | VUS  (PM2) |
| 109 | HCM | No | TNNT2 | NM_001001430 | c.857G>A, p.Arg286His | Missense | HTZ | HCM (DM) /Novel | . | 7.88E-05 | VUS  (PP5) |
| 79 | HCM | YES | TPM1 | NM_001018005 | c.523G>A,  p.Asp175Asn | Missense | HTZ | HCM (DM)/ P | . | . | VUS  (PM2;PP3;PP5) |
| 61 | DCM | Yes | DSP | NM_004415 | c.4997G>A p.Arg1666Gln | Missense | HTZ | Novel/VUS | . | 3.33E-05 | VUS  (PM2;BP1) |
| 68 | DCM | No | EYA4 | NM_004100 | c.978C>G, p.Phe326Leu | Missense | HTZ | NSHL(DM)  /VUS | . | 4.14E-05 | VUS  (BP1) |
| 63 | DCM | No | KLF10 | NM_005655 | c.1169C>T p.Thr390Met | Missense | HTZ | Novel/ Novel | 0.0002 | 5.43E-05 | VUS  (PM1) |
| 27 | DCM | No | LMNA | NM_170707 | c.169G>T p.Ala57Ser | Missense | HTZ | Novel/ Novel | . | . | VUS  (PM2) |
| 103 | DCM | No | LMNA | NM_170707 | c.1003C>T p.Arg335Trp | Missense | HTZ | DCM (DM)/ LP | . | . | VUS  (PM2;PP3;PP5) |
| 21 | DCM | No | NDUFV2 | NM_021074 | c.1A>G, p.Met1Val | Missense | HTZ | Novel/ Novel | . | . | VUS  (PM2) |
| 37 | DCM | No | NEBL | NM_006393 | c.1429G>C, p.Ala477Pro | Missense | HTZ | Novel/Novel | . | 1.65E-05 | VUS  (None) |
| 67 | DCM | No | PRKAG2 | NM_016203 | c.1657G>T, p.Ala553Ser | Missense | HTZ | Novel/ Novel | . | . | VUS  (PM1;PM2;PP3) |
| 87 | DCM | No | RBM20 | NM_001134363 | c.3545G>A, p.Arg1182His | Missense | HTZ | DCM (DM)/ conflicting interpretations | 0.0006 | 0.0001 | VUS  (PM1;PP3;PP5) |
|  |  |  | SCN5A | NM_198056 | c.2962C>T, p.Arg988Trp | Missense | HTZ | BrS (DM)/VUS | . | 3.96E-05 | VUS  (PM1;PM2) |
| 112 | DCM | No | TNNI3 | NM_000363.4 | c.416A>C p.Phe139Cys | Missense | HTZ | Novel/Novel | . | . | VUS  (PM2;PP3) |
| 93 | RCM | No | LMNA | NM_170707 | c.1682T>A p.Leu561Gln | Missense | HTZ | Novel/Novel | . | . | VUS  (PM1;PM2) |
| 94 | RCM | Yes | MYH7 | NM_000257 | c.1265A>G, p.Tyr422Cys | Missense | HTZ | Novel/Novel | . | . | VUS  (PM2;PP3) |
| 91 | RCM | No | MYH7 | NM_000257 | c.2302G>A, p.Gly768Arg | Missense | HTZ | HCM (DM) )/ P | . | . | VUS  (PM2;PP3;PP5) |
| 20 | RCM | No | PSEN1 | NM_000021.3 | c.118G>A, p.Asp40Asn | Missense | HTZ | Novel/ Novel | . | 8.26E-06 | VUS  (PM2;PP3) |
| 105 | RCM | No | SCN5A | NM_198056.2 | c.3521G>C, p.Arg1174Pro | Missense | HTZ | Novel/ Novel | . | . | VUS  (PM1;PM2;PP3) |
| 98 | RCM | No | SCN5A | NM_198056 | c.3556G>A, p.Ala1186Thr | Missense | HTZ | LQTS(DM)/VUS | . | 2.45E-05 | VUS  (PM1;PM2;PP5;BP4) |
| 80 | RCM | No | TNNI3 | NM_000363.4 | c.610C>T，p.Arg204Cys | Missense | HTZ | HCM (DM) / conflicting interpretations | . | . | VUS  (PM2;PP3;PP5) |
| 7 | RCM | No | TNNT2 | NM_001001430.2 | c.281G>T p.Arg94Leu | Missense | HTZ | HCM (DM) / VUS | . | . | VUS  (PM2;PP3;PP5) |
| 11 | LVNC | No | DSG2 | NM_001943 | c.178G>A p.Glu60Lys | Missense | HTZ | ARVC(DM?)/ VUS | . | . | VUS  (PM2;PP3) |
| 16 | LVNC | No | DSG2 | NM_001943 | c.2906C>T p.Ala969Val | Missense | HTZ | Novel/ conflicting interpretations | 0.0008 | 0.0005 | VUS  (PM2;PP3;BS2) |
| 72 | LVNC | No | DSP | NM_004415 | c.5878A>C p.Thr1960Pro | Missense | HTZ | Novel/ Novel | . | . | VUS  (PM2;BP4) |
| 88 | LVNC | No | DSP | NM_004415 | c.8125G>A p.Ala2709Thr | Missense | HTZ | Novel/ Novel | . | . | VUS  (PM2) |
| 117 | ARVC | No | MYH6 | NM_002471 | c.2306C>T,p.Ala769Val | Missense | HTZ | Novel/ Novel | . | 5.00E-05 | VUS  (PM1;PP3) |
| 104 | ARVC | No | MURC | NM_001018116 | c.316C>T p.Arg106Trp | Missense | HTZ | Novel/ Novel | 0.0004 | 0.0001 | VUS  (None) |
| 44 | Undefined Cardiomyopathy | No | ACTN2 | NM_001103.3 | c.1586A>G p.Asn529Ser | Missense | HTZ | Novel/ VUS | . | 4.12E-05 | VUS  (None) |
| 52 | DCM+muscular weakness | No | DSG2 | NM_001943 | c.1829T>C p.Leu610Pro | Missense | HTZ | Novel/ Novel | . | 2.49E-05 | VUS  (PM2) |
| 53 | HCM+DCM | No | DSP | NM_004415 | c.4702_4707delCTGCAG p.1568_1569delLeuGln | Inframe deletion | HTZ | Novel/ Novel |  |  | VUS  (PM2;PM4) |
| 55 | myopathy+DCM | No | KLF10 | NM_005655 | c.80T>C p.Met27Thr | Missense | HTZ | HCM (DM) / Novel | . | 8.24E-05 | VUS  (BP4) |
| 36 | DCM,  AF, VT | No | MYH7 | NM_000257 | c.5656-4G>A | Splicing | HTZ | Novel/ Novel | . | 4.12E-05 | VUS  (BP4) |
| 47 | LVNC or DCM | No | NEBL | NM_006393 | c.515C>A, p.Thr172Lys | Missense | HTZ | Novel/ Novel | 0.0002 | 1.65E-05 | VUS  (BP4) |
| 26 | DCM or LVNC | No | PSEN2 | NM_000447.2 | c.505C>A, p.His169Asn | Missense | HTZ | Alzheimer disease(DM)/ Novel | 0.0002 | 0.0002 | VUS  (PP3) |
| 116 | Undefined Cardiomyopathy | No | TNNT2 | NM_001001430 | c.745G>A, p.Asp249Asn | Missense | HTZ | Novel/ conflicting interpretations | 0.0002 | 1.65E-05 | VUS  (PP3;PP5;BS2) |
| 66 | DCM，muscular myopathy | No | VCL | NM_014000 | c.1273C>G, p.Pro425Ala | Missense | HTZ | Novel/ Novel | . | . | VUS  (PM2) |
| 64 | Undefined Cardiomyopathy | No | VCL | NM_014000 | c.884G>A, p.Gly295Asp | Missense | HTZ | Novel/ Novel | . | . | VUS  (PM2;PP3) |
| 92 | HCM?  DCM? | No | VCL | NM_014000 | c.2630C>T, p.Pro877Leu | Missense | HTZ | Novel/ Novel | 0.0002 | 2.47E-05 | LB  (BS2) |
|  |  |  | PSEN2 | NM_000447.2 | c.998A>G, p.Glu333Gly | Missense | HTZ | Novel/ Novel | . | . | VUS  (PM2;PP3) |
| 56 | HCM | No | BAG3 | NM_004281 | c.772C>T p.Arg258Trp | Missense | HTZ | Prolonged QT interval (DM?)/ Conflicting interpretations of pathogenicity | 0.0042 | 0.0012 | LB  (PP3;PP5;BS1;BP6) |
|  |  |  | VCL | NM_014000 | c.133G>T, p.Ala45Ser | Missense | HTZ | Novel/ Novel | . | . | VUS  (PM2) |
| 114 | HCM | No | MYBPC3 | NM_000256 | c.1000G>A, p.Glu334Lys | Missense | HTZ | HCM (DM)/ conflicting interpretations | 0.001 | 0.0003 | LB  (PM2;PP5;BS2;BP6) |
| 30 | ARVC | No | MYBPC3 | NM_000256 | c.2992C>G, p.Gln998Glu | Missense | HTZ | HCM (DM)/ conflicting interpretations | 0.0062 | 0.0052 | B  (PM1;PP3;PP5;BS1;BS2;BP6) |

Note: HGMD: for Human Gene Mutation Database; DM, for damaging-mutation. BrS: Brugada syndrome; NSHL: Sensorineural hearing loss, nonsyndromic; LQTS: Long QT syndrome; VUS: Uncertain significance variants; AF: [atrial](../../../../C:/Users/Genetics/AppData/Local/youdao/Dict/Application/6.3.69.5012/resultui/frame/javascript:void(0)%3B) [fibrillation](../../../../C:/Users/Genetics/AppData/Local/youdao/Dict/Application/6.3.69.5012/resultui/frame/javascript:void(0)%3B); VT: ventricular tachycardia. HTZ: Heterozygous. B: benign; LB: likely benign; P: Pathogenic; LP: Likely pathogenic.
